# Genotype by Sex and Genotype by Age Interactions with Sedentary Behavior: The Portuguese Healthy Family Study

**DOI:** 10.1371/journal.pone.0110025

**Published:** 2014-10-10

**Authors:** Daniel M. V. Santos, Peter T. Katzmarzyk, Vincent P. Diego, John Blangero, Michele C. Souza, Duarte L. Freitas, Raquel N. Chaves, Thayse N. Gomes, Fernanda K. Santos, José A. R. Maia

**Affiliations:** 1 Centre of Research, Education, Innovation and Intervention in Sport, Faculty of Sports, University of Porto, Porto, Portugal; 2 Pennington Biomedical Research Center, Louisiana State University System, Baton Rouge, Louisiana, United States of America; 3 Texas Biomedical Research Institute, San Antonio, Texas, United States of America; 4 Sport and Physical Education Department, University of Madeira, Funchal, Portugal; 5 Physical Education Department, Federal University of Technology - Parana, Campus Curitiba, Curitiba/PR, Brasil; INIA, Spain

## Abstract

Sedentary behavior (SB) expression and its underlying causal factors have been progressively studied, as it is a major determinant of decreased health quality. In the present study we applied Genotype x Age (GxAge) and Genotype x Sex (GxSex) interaction methods to determine if the phenotypic expression of different SB traits is influenced by an interaction between genetic architecture and both age and sex. A total of 1345 subjects, comprising 249 fathers, 327 mothers, 334 sons and 325 daughters, from 339 families of The Portuguese Healthy Family Study were included in the analysis. SB traits were assessed by means of a 3-d physical activity recall, the Baecke and IPAQ questionnaires. GxAge and GxSex interactions were analyzed using SOLAR 4.0 software. Sedentary behaviour heritability estimates were not always statistically significant (p>0.05) and ranged from 3% to 27%. The GxSex and GxAge interaction models were significantly better than the single polygenic models for TV (min/day), EEsed (kcal/day), personal computer (PC) usage and physical activty (PA) tertiles. The GxAge model is also significantly better than the polygenic model for Sed (min/day). For EEsed, PA tertiles, PC and Sed, the GxAge interaction was significant because the genetic correlation between SB environments was significantly different from 1. Further, PC and Sed variance heterogeneity among distinct ages were observed. The GxSex interaction was significant for EEsed due to genetic variance heterogeneity between genders and for PC due to a genetic correlation less than 1 across both sexes. Our results suggest that SB expression may be influenced by the interactions between genotype with both sex and age. Further, different sedentary behaviors seem to have distinct genetic architectures and are differentially affected by age and sex.

## Introduction

Sedentary behaviors, i.e. sitting or reclining, have been increasingly linked with poor health status, and are associated with chronic diseases such as type 2 diabetes, and overall mortality [1]. Furthermore, there is reliable evidence showing that the hazardous effects of sedentary lifestyles are observed across different age groups and in both sexes [1]–[3]. In a recent 5-year follow-up study it was observed that adolescents who engaged in excessive screen viewing had significantly lower quality of life scores [2]. In addition, evidence from longitudinal studies during adulthood show associations of sedentary behavior with site-specific cancers (ovarian, endometrial and colon), cardiovascular disease and type 2 diabetes [1]. During later life, some sedentary behaviors, such as television viewing, have been linked with lower cognitive performance [3].

In a review of the genetic basis of physical activity and sedentary behavior, Santos et al. [4] observed heritability estimates for sedentary behavior and physical inactivity phenotypes ranging from 0.00 in twins [5] to 0.60 in nuclear families [6]. Recently, in a sample of 1654 middle-aged twins, den Hoed et al. [7] found that genetic factors explained 31% of the variance in time spent in sedentary behaviors. Further, Simonen et al. [8] reported a heritability estimate of 25% in Canadian nuclear families for physical inactivity [9]. Given that there are few such studies focusing on the heritability of sedentarism, the exploration of the genetic architecture of different indicators of sedentary behavior in nuclear families will add to the ongoing debate about the regulation of sedentarism. Furthermore, information about sex and age effects on the genetic regulation of sedentary behavior is almost nonexistent.

The main purposes of the present study, using a nuclear family design, are to estimate the magnitude of genetic factors responsible for the variation in distinct sedentary behavior phenotypes, and to study potential Genotype-by-Sex and Genotype-by-Age interactions influencing these sedentary behaviors. We hypothesize that SB is genetically driven and that GxSex and GxAge interactions will have a significant influence in SB phenotype expression.

## Materials and Methods

*The Healthy Family Study*, from the Portuguese *Famílias Saudáveis* (FAMS), investigates the relationship among metabolic syndrome indicators, physical activity, physical fitness and body composition in nuclear families. Children and adolescents aged ≤ 18 years were recruited in schools from Azores and Madeira archipelagos, and the northern and central regions of mainland Portugal, and were approached to freely participate in the study with their siblings and parents. School officers provided family lists, and families with at least two siblings were initially invited. However, given that families with 3 or more children are scarce in the Portuguese population [10], and to improve statistical power, one-offspring families were randomly ascertained with respect to phenotype and recruited to the study. Subjects with chronic diseases, physical handicaps or psychological disorders were excluded as these conditions might impair their daily routines, namely their physical activities within schools and/or sports clubs. The ethics committee of the College of Sport, University of Porto, approved the study, and written informed consent was obtained from all subjects including the legal representatives of the children involved.

### Data Collection

The standardized procedures of Lohman et al. [11] were used to measure height with a Siber Hegner anthropometer (GMP instruments), and weight with a Tanita scale (model BC-418 MA).

Using a 3-d physical activity diary (B3DAR) [9], a trained technician interviewed each subject, recording the dominant activity for each 15-min period during 24 h by using a list of categorized activities. Categories from 1 to 9 refer to increasing levels of energy expenditure (METs) of each activity. Sedentary behaviors, such as sleeping or sitting, are listed in categories 1 to 3 and indicate low/very low METs. The number of 15-min periods for these three categories was first summed over the 3-day period and divided by 3 which resulted in minutes in sedentary behavior per day (Sed). Total energy expenditure in sedentary behaviors (TEESB) was then calculated by weighting each category for its own approximate median energy cost (kcal/kg/15 min). This value was then summed and multiplied by the subjectś body weights. Total daily sedentary energy expenditure [EEsed (kcal/day)] was then calculated by dividing TEESB by 3.

The Baecke questionnaire is a self-administered questionnaire aiming to analyze 3 different dimensions of habitual physical activity: (i) sports physical activity (SPA), (ii) leisure physical activity (LPA) and (iii) work/school physical activity (WPA). It is a Likert scale-based questionnaire that refers to the past 12 months, and includes 16 questions divided into 3 distinct sections: questions 1-8 assess work or school physical activity; questions 9–12 assess sports activities and exercise programs; and questions 13–16 assess leisure and locomotive activities. The sums of the specific scores of each section constitute the physical activity level for WPA, SPA and LPA, respectively. The sum of these 3 scores constitutes the habitual or total physical activity level (TPA). Sports were subdivided into three levels of energy expenditure according to Durnin and Passmore [12]; the low level for sports such as, billiards, sailing, bowling, golf (average energy expenditure 3 kcal/min); the middle level for sports such as, badminton, cycling, dancing, swimming and tennis (average energy expenditure 5 kcal/min); and the high level for sports such as boxing, football, basketball, rugby and rowing (average energy expenditure 7 kcal/min). A question about the total time per day the subject used a personal computer (PC) was applied to further classify sedentary behavior. Also, subjects were considered sedentary, light active and high active, depending upon if they were in the first, second, or third tertile of TPA.

The International Physical Activity Questionnaire (IPAQ) [13] was conceived to obtain comparable estimates of PA across countries. We applied the IPAQ short version (IPAQs), which is suitable for use in national and regional surveillance systems. Subjects were asked to recall the time spent in vigorous (activities in which the heart rate increases dramatically), moderate (activities in which the heart rate increases moderately, except walking) and low (activities such as walking) intensity activities performed in the last seven days at home/work, during leisure time and transportation. For this particular paper, IPAQ was used to assess time (minutes per day) watching TV (TV) and sitting.

### Statistical Analysis

#### Descriptive analysis

Descriptive analyses were performed in PEDSTATS v0.6.12 and group comparisons were made in PEPI 4.0.

#### Univariate genetic procedures

Univariate quantitative genetic procedures as implemented in SOLAR v4.1 [14] under a special class of the multivariate linear model, namely the variance components (VC) approach, were used to estimate additive genetic and environmental VCs for each of the SB traits. Prior to all modeling, age, age-squared, sex, age-by-sex, and age-squared-by-sex were used as covariates in a preliminary VC model. Residuals were thus derived for each trait and were normalized using an inverse normal transformation, as previously advocated [15], [16]. Assuming that dominance and epistasis are negligible, comparisons of individual phenotype *y* define the covariance of the basic polygenic model as:
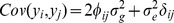
(Eq.1)where *i* and *j* index individuals, 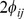 gives the expected coefficient of relationship, 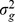 is the additive genetic variance, 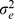 is the environmental variance, and 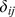 isdefined as 1 when individuals *i* and *j* are the same, and 0 otherwise. This model is used to estimate heritability, which is given as: 
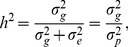
(Eq.2)where 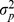 is the total phenotypic variance. Heritability estimates (h^2^) were computed using a maximum likelihood approach to estimate variance components under the standard polygenic model as implemented in SOLAR v.4.3.1 software [14].

#### Gene-by-Sex and Gene-by-Age interactions

To test for GxSex and GxAge interactions, basic initial hypotheses were formulated regarding the variance/covariance relationship of a SB indicator between family members with different gender or age. For the GxSex model we treated sex as a discrete environment (male or female), and for the GxAge model we treated the age continuum as a continuous environment. As regards Genotype by Environment (GxE) interaction in general, the fundamental null hypothesis is that the expression of a polygenotype (i.e., aggregate of all genotypes related to the expression of a phenotype) is independent of the environment. It can be shown from first principles that if there is no GxSex or GxAge interaction, the same SB trait measured in subjects with different genders or ages will have a genetic correlation of 1.0 and the genetic variance will be homogeneous across both genders or across all ages [17], [18]. On the contrary, if GxSex or GxAge interactions are present, the genetic correlation will be significantly less than 1.0 and/or the genetic variance will not be the same in different genders or ages.

The basic polygenic model can be extended to account for GxSex interaction by allowing for sex-specific additive genetic and environmental variances denoted by 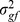, 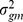, 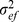, and 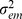, where *f* and *m* index females and males, respectively, and for an across-sex genetic correlation denoted by 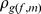. Under the GxSex model, the two null hypotheses are 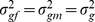 and 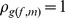.

Under the GxAge interaction model, variance and correlation are modeled as continuous functions of age. For the genetic variance function (and similarly for the environmental variance function) variance is modeled using an exponential function to ensure positivity, which is required since any variance is a squared term [17], [18]:
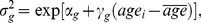
(Eq.3)where 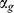 and 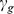 are parameters to be estimated, and 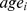 is the age of the *i*-th individual standardized against the sample mean,. An additional justification for the exponential function is suggested by the alternative name of this approach, namely the log-linear model of the variance: 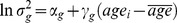
(Eq.4)


That is, on taking the natural logarithm of the variance modeled as an exponential function, we have the equation of a line. In this form, the variance homogeneity null hypothesis obviously holds for a slope-term equal to 0: 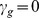. For the genetic correlation function, we modeled the genetic correlation as an exponential decay function of the pair-wise age differences for the *i*-th and *j*-th individuals: 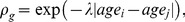
(Eq.5)where 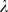 is a parameter to be estimated. Here we also have an elegant re-expression of the interaction null hypothesis, in this case regarding the genetic correlation, in that a genetic correlation equal to 1 is equivalent to 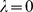. This is because for 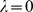, we have 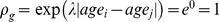.

For reasons detailed in Diego et al. [17], the likelihood ratio test statistic (LRT) to test 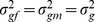 or 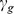 under the GxSex or GxAge interaction models is distributed as 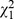, a chi-square random variable with 1 degree of freedom (d.f.), and the LRT to test 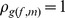 and 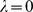 is distributed as 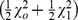, a 50∶50 mixture of chi-square random variable with a point-mass at 0, denoted by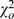, and a chi-square with 1 d.f. Prior to examination of these hypotheses, we first confirmed if the overall GxSex and GxAge interaction models provided a better fit to the data than the standard so-called polygenic models. The LRT for this comparison can be shown to be distributed as 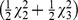 [19].

We also performed a post-hoc power analysis to determine the power in our sample to detect GxSex and GxAge interaction effects. Power is defined as the probability of correctly rejecting the null hypothesis, and is computed as the probability integral from the point on the alternative distribution corresponding to the nominal significance level or alpha (on the null distribution) to the upper limit of the alternative distribution at positive infinity. Since the total probability of any distribution is 1, power can be conveniently computed as:


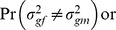
 
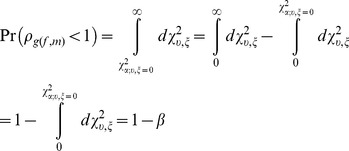
 (Eq.6) where the distribution under the alternative hypothesis is the non-central chi-square distribution, denoted by 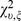, 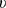 is the degrees of freedom (d.f.) parameter, 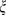 is the non-centrality parameter (NCP), 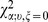 is the point on the non-central chi-square distribution corresponding to the 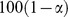 percentage point on the distribution under the null hypothesis, and 
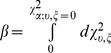
(Eq. 7) is the probability of making a type II error. We used a similar approach to that used by Blangero et al. [15].

## Results

Table 1 presents basic descriptive information. Some relatives were not able to fully engage in the data collection procedures (fathers = 32.5%; mothers = 3.6%; sons = 1.5%; daughters = 4.1%). As such, a total of 1345 subjects, comprising 249 fathers, 327 mothers, 334 sons and 325 daughters, from 339 families were included. The average family size was 3.97 subjects.

**Table 1:** Sample descriptive statistics.

		Father	Mother	Son	Daughter
		Mean ± Std.Dev.	Mean ± Std.Dev.	Mean ± Std.Dev.	Mean ± Std.Dev.
Age (yrs)		45.93±4.38	43.55±4.41	14.68±2.85	14.33±2.84
Height (cm)		170.39±6.67	158.62±5.76	162.41±13.36	155.93 ± 9.85
Weight (kg)		80.19±12.73	67.22±10.23	57.69±16.83	52.81±12.86
BMI (kg/m^2^)		27.62±3.98	26.72±3.92	21.47±4.23	21.47±3.99
Sitting (min/day)		291.26±211.77	306.85±284.20	345.06 ± 273.54	364.07±218.09
TV (min/day)		97.85±±75.14	75.74 ± 59.20	137.35±130.01	113.47±105.65
Sed (min/day)		1040.85±186.28	950.80±180.26	1233.14±112.22	1232.21±110.25
EEsed (kcal/day)		2099.57±536.17	2063.79±485.44	1583.31±479.51	1485.95±386.64
		%	%	%	%
PC	<30 min	39.7%	43.8%	16.5%	25.8%
	30 min – 60 min	13.8%	14.3%	27.0%	27.2%
	60 min – 90 min	6.9%	5.8%	20.9%	19.8%
	90 min – 120 min	5.8%	5.4%	15.2%	11.1%
	> 120 min	33.9%	30.8%	20.4%	16.1%

Legend: TV – television viewing; Sed – minutes spent in sedentary behaviors as assessed by B3DAR; EEsed – energy expenditure in sedentary behaviors as assessed by B3DAR; PC – daily personal computer usage (ordinal variable).

As assessed by the Baecke questionnaire, no differences were observed for PC usage between fathers and mothers (χ^2^ = 0.401, p = 0.05), sons and daughters (χ^2^ = 3.322, p = 0.07), and between parents and offspring (χ^2^ = 3.413, p = 0.07). As for the IPAQ phenotypes, offspring spend more time watching TV (p<0.01) and sitting (p<0.001) than parents. Mothers spend less time watching TV (p<0.01) and no significant differences were observed between fathers and mothers sitting time. Parents' energy expenditure in SB is higher than offsprings' (p<0.001), even though time spent in sedentary behaviors as assessed by the B3DAR was higher in offspring (p<0.001).

Heritability estimates presented in Table 2 varied in their magnitude (from 0.03 to 0.27) and significance. Time spent in sedentary behaviors was the most heritable trait with a value of 27%. followed by PA tertiles with and heritability estimate of 23%. On the other hand. PC usage assessed in an ordinal 5 point scale. sitting time (min) and TV watching (min) from the IPAQ were not significantly heritable.

**Table 2:** Heritability estimates (h^2^) and % of variance accounted for by covariates of different sedentary phenotypes in the Portuguese Healthy Families Study.

Trait	h^2^	Std. Error	p-value	% variance accounted for by covariates^1^
TV (min/day)	0.05	0.06	0.235	6.00
Sed (min/day)	0.27	0.06	<0.001*	41.00
EEsed (kcal/day)	0.19	0.07	0.003*	33.00
PC	0.04	0.07	0.265	1.00
Sitting (min/day)	0.03	0.09	0.344	1.00
PA Tertiles	0.23	0.06	<0.001*	0.00

Significant estimates are labeled with an *.

Legend: Tv – television viewing; Sed – minutes spent in sedentary behaviors as assessed by B3DAR; EEsed – energy expenditure in sedentary behaviors as assessed by B3DAR; TV – television viewing (ordinal variable); PC – personal computer usage (ordinal variable).

1 Covariates - age, age^2^, sex and their interactions.

To test for the influence of age and sex on the expression of SB indicators, the polygenic model was compared to its alternative models (GxSex and GxAge) by means of a log-likelihood ratio test (see Table 3). The GxSex and GxAge interaction models are significantly better than the polygenic model for TV (min/day), EEsed (kcal/day), PC (ordinal variable) and PA tertiles (see Table 4). Further, GxAge is also significantly better than the polygenic model for Sed (min/day). These results only mean that the GxSex and GxAge interaction models fit the data better than the polygenic model for these SB traits.

**Table 3:** Results of log-likelihood ratio tests (LRT) and respective p-values contrasting a polygenic model vs a GxSex model for each of the SB indicators.

Trait	Polygenic LnL	GxSex LnL	LRT	p-value
TV (min/day)	−358.811	−346.953	23.715	<0.001*
Sed (min/day)	−457.404	−456.522	1.764	0.302
EEsed (kcal/day)	−457.592	−453.058	9.066	0.009*
PC	−421.924	−417.977	7.895	0.015*
Sitting (min/day)	−338.818	−337.030	3.577	0.124
PA Tertiles	−489.192	−468.144	42.097	<0.001*

Significant estimates are labeled with an *.

Legend: TV – television viewing; Sed – minutes spent in sedentary behaviors as assessed by B3DAR; EEsed – energy expenditure in sedentary behaviors as assessed by B3DAR; PC – personal computer usage (ordinal variable).

**Table 4:** Results of log-likelihood ratio tests (LRT) and respective p-values contrasting a polygenic model vs a GxAge model for each of the SB indicators.

Trait	Polygenic LnL	GxAge LnL	LRT	p-value
Tv (min/day)	−358.811	−329.454	58.715	<0.001*
Sed (min/day)	−457.404	−396.015	122.778	<0.001*
EEsed (kcal/day)	−457.592	−429.013	57.157	<0.001*
PC	−421.924	−406.722	30.405	<0.001*
Sitting (min/day)	−338.818	−336.627	4.383	0.167
PA Tertiles	−489.192	−481.619	15.147	0.001*

Significant estimates are labeled with an *.

Legend: TV – television viewing; Sed – minutes spent in sedentary behaviors as assessed by B3DAR; EEsed – energy expenditure in sedentary behaviors as assessed by B3DAR; PC – personal computer usage (ordinal variable).

In order to assess if there is GxAge and/or GxSex interactions, each of the full models were compared to its constrained alternatives (i.e. setting 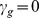 or 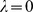 for GxAge and 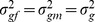 or 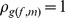 for GxSex) for Tv, Sed, EEsed, PC, sitting and PA tertiles.

**Figure 1:**
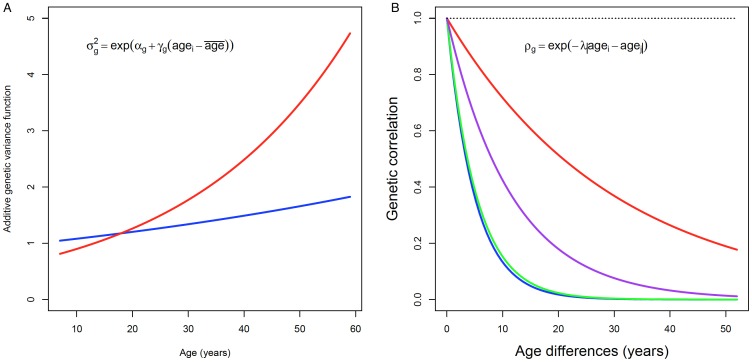
Genotype X Age genetic variance (a) and Genotype X Age genetic correlation (b). Legend: Blue – PC; Red – Sed; Green – EE; Purple – PA tertiles.

Figure 1 shows the results for those traits (PC, Sed, EE and PA tertiles) that showed significant variance heterogeneity and a correlation function that is significantly different from 1 in the GxAge model. For EEsed and PA tertiles significant GxAge interaction was due to rejection of the genetic correlation hypothesis (p<0.05). For PC and Sed, both null hypotheses, i.e., genetic correlation (ρ_g_) equals 1 and variance homogeneity, were significantly rejected. Figure 1a highlights that, for PC and Sed genetic variance increases with age. Concomitantly, Figure 1b displays that, for EEsed, PA tertiles, PC and Sed, the genetic correlation decreases with increasing differences between family members' ages.

**Figure 2:**
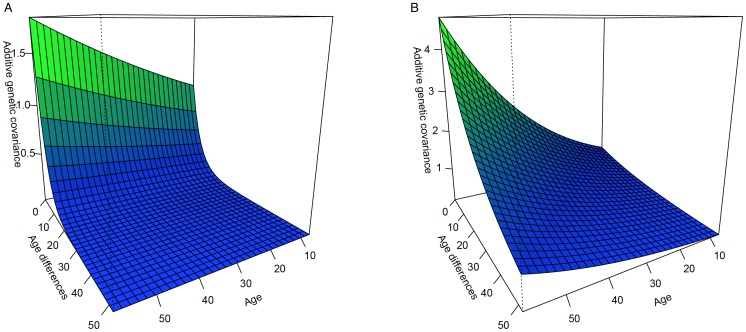
Genotype X Age for PC (A) and Sed (B).

Figure 2 illustrates that GxAge interaction for PC and Sed, is a joint function of genetic variance heterogeneity and a genetic correlation function not equal to one. Thus, we express them jointly as a covariance function in the vertical axis.

**Figure 3:**
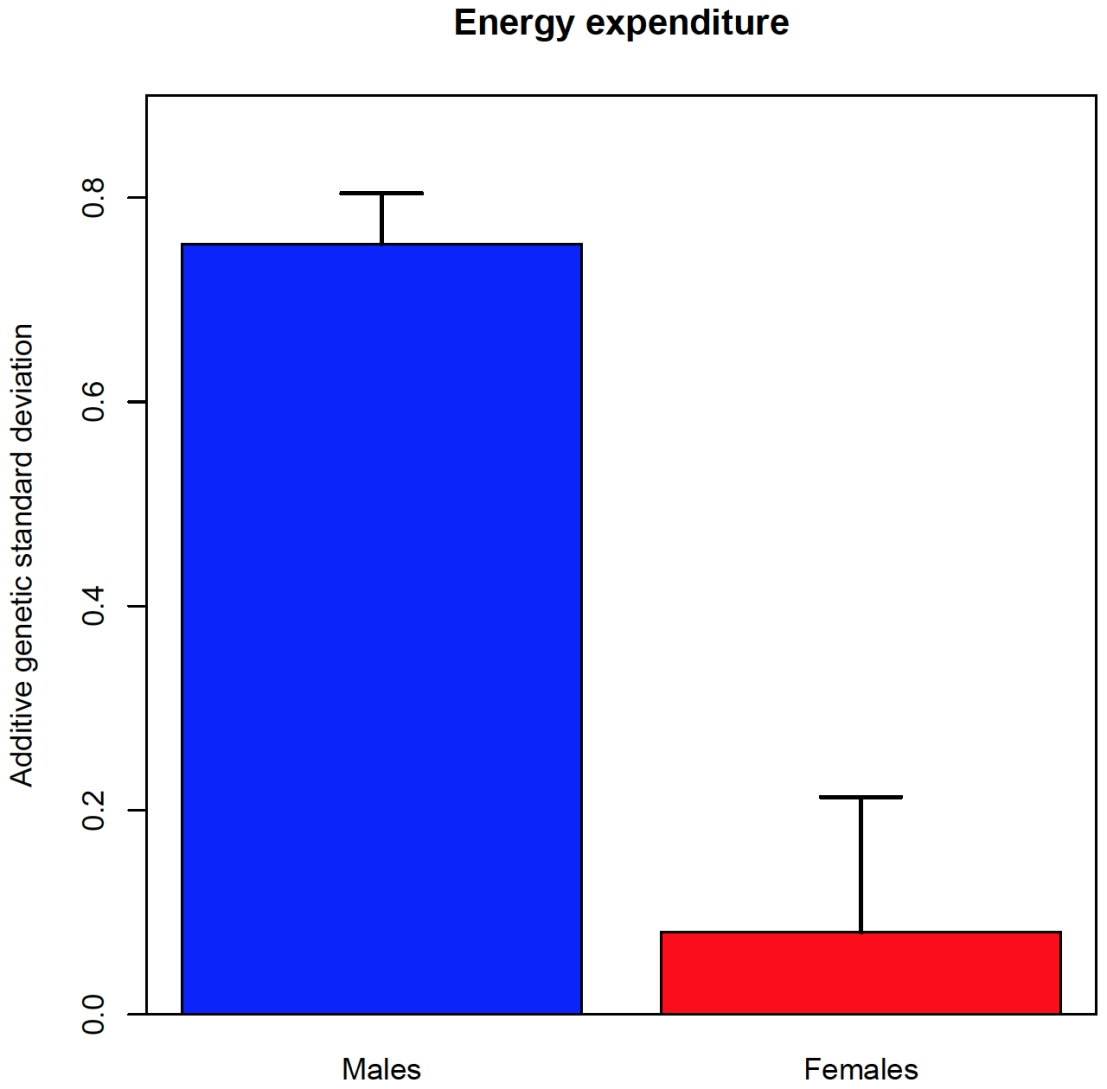
Genotype X Sex genetic variance for EEsed.

Figure 3 shows that the additive genetic effects in EEsed is greater in males than in females, suggesting that the expression of EEsed is under more genetic control in boys than in girls. That is, there is significant GxSex interaction for EEsed due to genetic variance heterogeneity. We also found significant GxSex interaction for PC due to a genetic correlation less than 1 across the sexes (ρ_g_ = −0.85; p = 0.009).

The power analysis results for the GxSex model are presented in Table 5. Taking 80% power as the criterion for sufficient power to detect a GxSex effect, we found that we did not have sufficient power in the sample for any of the traits. However, power is highest for the two traits for which at least one of the null hypotheses were rejected. For EEsed we found evidence of GxSex interaction due to variance heterogeneity, and the power to detect GxSex interaction due to variance heterogeneity is highest for this trait. Similarly, for PC we found evidence of GxSex interaction due to a genetic correlation less than 1, and the power to detect this particular interaction effect was highest for PC.

**Table 5:** Power to detect GxSex interaction effects.

Trait	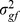	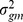	Power	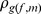	power
EEsed	0.08066	0.75473	0.72809	1	0.1
PC	0.53845	0.53375	0.05008	−0.84644	0.77167
Sed	0.63816	0.39684	0.21	0.97973	0.10011
Sit	0.30612	0.01312	0.0801	1	0.1
PA tertiles	0.38023	0.47452	0.07131	0.86639	0.10578
TV	0.20082	0.63	0.36311	0.98777	0.10001

The power analysis results for the GxAge model are presented in Table 6. Unlike our power to detect GxSex interaction effects, we had for the most part ample power to detect GxAge interaction effects in regard to rejection of both null hypotheses of variance homogeneity and a genetic correlation equal to 1. It should be noted that sufficient power to reject either null hypothesis when they are false does not necessarily mean that they are false.

**Table 6:** Power to detect GxAge interaction effects.

Trait	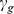	power	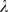	Power
EEsed	0.02159	1	0.12883	1
PC	0.0107	0.90895	0.20179	1
Sed	0.03388	1	0.03327	1
Sit	−0.00512	0.96473	0.63717	0.9894
PA tertiles	−0.01939	1	0.08582	0.79523
TV	0.00651	1	0.03306	1

## Discussion

The novelty of these results presented in this study is the estimation of heritability for a set of distinct SB markers in nuclear families as well as the application of genotype x environment interaction models to better understand how SB is influenced by age and sex. It should be emphasized that the heritabilities varied from 3% (sitting) to 27% (Sed), which means that these phenotypes have distinct genetic contributions. This is quite relevant to understanding the mechanisms that lead to distinct sedentary behaviors and suggests that even though some behaviors are under some genetic regulation, others seem to be just an adaptation to the contemporary way of living. The only result that could be compared with other studies is from the 3-d diary, that yielded an heritability estimate of 27% for sedentary behavior which is in agreement with previous data from the Quebec Family Study [8] showing that 25% of the variance in the same phenotype was due to genetic factors.

Some studies have used accelerometry to quantify sedentary behavior [5], [20] in genetic studies. For example, the *Viva La Familia* study [20] demonstrated a heritability estimate of 57% for the percentage of wake time in activities with an energy expenditure of ≤ 0.01 kcal/kg/min. More recently, an adult twin study [5] yielded an heritability estimate of 0 for sedentary time defined as <100 counts/min. Further, a robust analysis of 1654 twins made by den Hoed et al. [7] showed that 35% of the variance in SB assessed by accelerometry and heart rate monitors was due to genetic factors. This obvious disparity in the results may be due to different sample sizes, design, distinct markers of SB and cut-points used and/or different statistical procedures and corresponding statistical adjustments for diverse covariates. However, we argue that these discrepancies may be related to an imprecise definition of SB and the use of distinct markers that may not measure the same thing. In fact, sedentarism has been defined as a group of behaviors that occur whilst sitting or lying down while awake and typically require very low energy requirements [21]. This definition has led to the emergence of several markers (e.g., television viewing, computer usage, time seated during work or in school) expressed in different units (e.g., minutes, METs, or ordinal scales) that map different facets of sedentarism, that may hinder our understanding of the putative mechanisms that regulate sedentary behaviors. Therefore, it may not be advisable to compare the heritability of different indicators, as these are not the same phenotypes and any comparison has a degree of associated bias.

The growing interest in the association between SB and health has led researchers to examine the influence of age and sex in the expression of distinct sedentary behaviors. Specifically, Genotype x Sex and Genotype x Age interaction analyses can be very helpful to disentangle these issues and are a sophisticated way to analyze the association of age and gender with SB. We found that the GxSex interaction model was a significantly better predictor of TV, EEsed, PC and PA tertiles than the polygenic model, meaning that there is variability in the expression of these behaviors that may be explained by differences between males and females in the genetic regulation of SB. Given the consistently higher additive genetic variance in males, our interpretation of these results is that the genetic regulation of energy expenditure in sedentary behaviors is stronger in males than in females. Once again, we were not able to find any study comparable to ours, namely because our model does not include any ‘family/shared’ factors as a source of environmental variation. However, previous work [22] has shown that the household effect under the polygenic model and under a similar GxSex model failed to be significant for any of their PA traits. Recently, in a sample from the Netherlands Twin Registry (NTR) (aged 12–20 y), SB was defined as an overall score of weekly frequency of television viewing, playing electronic games, and computer or internet use, and the results showed that SB was significantly higher for boys than girls (χ^2^ = 755.56; P<0.05), highlighting the presence of sex differences in the expression of SB. However, in 2010, another study with the NTR found that irrespective of age, sedentarism is more prevalent in females when using a distinct marker of SB (subjects were sedentary if total weekly MET score was lower than 5.0, i.e., the energy expenditure due to PA was lower than 1 kcal·kg^−1^·h^−1^). Once again, this seems to be quite difficult to understand, as even within the same sample and the same research group, SB results can be so diverse. This is why SB may not be a one-dimensional phenotype *per se*. Rather, it is a multi-dimensional behavior, characterized by low energy expenditure that is quite diverse in their structure, levels and patterns. Our results raise new questions regarding daily activities and/or routines, because evidence shows that males tend to be more active than females [23], [24]. So, if males are more prone to suffer from environmental exposures that lead to SB, how come they are more active than females? It has been shown that SB may coincide with sports activities. For instance, elite athletes present long periods of sedentariness in their daily routines while recovering from their highly intense training sessions [25]. In this case, we might speculate that the environment that leads males to more intense activities is also the force leading to increased levels of sedentarism.

In regard to the influence of age on SB, the GxAge interaction model was a significantly better predictor of TV viewing, EEsed, PC, Sed and PA tertiles than the polygenic model, meaning that the expression of SB could be age-specific. In this case, the age effect is not simply the accumulation of habits and experiences as people get older, but mainly the way by which our genetic background probably deals with those routines. To the best of our knowledge, this kind of analysis has never been performed on SB and is of utmost importance because it can help to explain why some people tend to become sedentary as age increases. Typically, it is expected that people become more sedentary as they move from childhood to adolescence, adulthood and finally late adulthood. This has been attributed to changes in lifestyle between these life stages. For instance, adolescent development is known for being highly dependent upon peer approval, as engaging in sports or leisure's activities is dependent upon being part of a team or club [26], [27], and is also influence by physical ability as being successful in a sport is a highly significant environmental factor contributing to increased levels of physical activity [28]. Moving to adulthood, people tend to spend more time seated due to their job responsibilities and during late adulthood people's low fitness levels determine the increase of sedentarism [29], [30]. However, this study attempts to understand if SB is not only a function of the environmental changes that most people (at least in developed countries) experience throughout a lifespan but also if those changes can be distinctly integrated by different subjects. The current study found that SB expression is influenced by a mediation effect of age through genetic regulation which might explain why some people are not sedentary even at older ages.

Some limitations of the present study must be addressed: (i) our sample is not representative of the general Portuguese population, (ii) the relatively young and healthy sample of families may limit the generalizability of the results to older individuals, (iii) the usage of self-reporting SB is doomed to diminished reliability when compared to more objective measures which may influence heritability estimates, and (iv) the lack of information about resting metabolic rate may influence the EEsed and Sed results, although previous work [31], [32] with this 3-d diary never considered this possibility. Further, the lack of sufficient power in the sample to estimate GxSex interactions for any of the traits is a serious weakness. Still, our sample is made of 339 families, which is in line with previous analysis performed with 319 families from the Viva la Familia Study (30) and 207 families from the Quebec Family Study [8]. Moreover, the reliance on different markers of SB, the use of state-of-the-art statistical procedures and the novelty of the analytical strategy are strengths of the present study.

In conclusion, the present results highlight that, in our sample, 3 to 27% of the variation in sedentary behaviors is due to a genetic component adding to the known fact that sedentarism is a multifaceted and variable construct that is highly dependent upon measurement strategy. Further, our results suggest that SB expression may be influenced by the interactions between genes with both sex and age, which helps to understand some of the observed variation in these traits' expression. However, it must be acknowledged that different sedentary behaviors have distinct genetic foundations and are affected differently by age and sex, which should motivate efforts to develop better assessment tools that can be more easily comparable.
